# A Model Design for Risk Assessment of Line Tripping Caused by Wildfires

**DOI:** 10.3390/s18061941

**Published:** 2018-06-14

**Authors:** Shuzhu Shi, Chunjing Yao, Shiwei Wang, Wenjun Han

**Affiliations:** 1 School of Remote Sensing and Information Engineering, Wuhan University, Wuhan 430079, China; shishuzhu@whu.edu.cn; 2 School of Electronic Information, Wuhan University, Wuhan 430072, China; wangsw@whu.edu.cn; 3 State Grid Economic and Technological Research Institute Co. Ltd., Beijing 102209, China; hanwenjun730@163.com

**Keywords:** high-voltage power line corridors, line tripping, logic regression, risk assessment, wildfire occurrence

## Abstract

A power line is particularly vulnerable to wildfires in its vicinity, and various damage including line tripping can be caused by wildfires. Using remote sensing techniques, a novel model developed to assess the risk of line tripping caused by the wildfire occurrence in high-voltage power line corridors is presented. This model mainly contains the wildfire risk assessment for power line corridors and the estimation of the probability of line tripping when a wildfire occurs in power line corridors. For the wildfire risk assessment, high-resolution satellite data, Moderate Resolution Imaging Spectroradiometer (MODIS) data, meteorological data, and digital elevation model (DEM) data were employed to infer the natural factors. Human factors were also included to achieve good reliability. In the estimation of the probability of line tripping, vegetation characteristics, meteorological status, topographic conditions, and transmission line parameters were chosen as influencing factors. According to the above input variables and observed historical datasets, the risk levels for wildfire occurrence and line tripping were obtained with a logic regression approach. The experimental results demonstrate that the developed model can provide good results in predicting wildfire occurrence and line tripping for high-voltage power line corridors.

## 1. Introduction

With fast economic growth in China, the demand for electric power has increased significantly, resulting in rapid progress in development of power infrastructure. Many high-voltage power transmission networks, which have a total length of more than 100,000 km, have been built to transfer electric power from the northwest and southwest regions to the relatively developed central and eastern regions due to the imbalanced distribution of energy resources and loads in China [1]. On the other hand, the rapid development of high-voltage power transmission networks has increased the requirements of land occupation. To resolve this issue, large parts of the power transmission networks are located inside forested land and grassland. Furthermore, due to the implementation of the Grain for Green Project in China since 1999 [2], not only total forest area but also vegetation density in the vicinity of high-voltage power transmission networks have obviously increased. As a result, the recorded number of line trippings caused by wildfires continues to grow, and wildfire has been regarded as one of the main threats to the safe and reliable operation of high-voltage power transmission networks [3,4].

Due to the negative impacts of wildfire, numerous studies have been conducted to assess the risk of its occurrence. Among existing studies, both natural and human factors were employed as input variables for evaluating fire danger such as meteorological conditions, topographic status, vegetation characteristics, and anthropogenic parameters [5]. Furthermore, a variety of methods were used for estimating wildfire risk such as a logistic regression model [6], a fuzzy set theory integrated with a decision-making algorithm [7], a Random Forest [8], and an artificial neural network [9]. In addition, different experiments were performed to evaluate the performance of developed models. For example, Satir et al. [9] employed the relative operating characteristic analysis to validate prediction results, and declared that the coefficient of accuracy of their model was 0.83 when it was used for mapping forest fire probability in a Mediterranean forest ecosystem. Zhang et al. [10] used the area under the curve (AUC) value to evaluate the developed model’s predictive performance, and showed that their model had 94.4% accuracy when it was applied to the prediction of forest fire risk in Northeast China. Although some good results have been obtained for early warning of wildfires, a generalized wildfire risk model that can provide accurate prediction results over a broad range of geographic areas remains a challenging task because of the complex relationships between fire occurrence and influencing factors [11]. Different from most existing works on predicting forest fire risk where the natural factors have a larger contribution, human activities play the most important role in the wildfire occurrence in high-voltage power line corridors (PLCs) according to these analyses described in [3,4]. Among existing studies, Lu et al. [12] and Chen et al. [13] exploited remote sensing and geographic information system (GIS) techniques for wildfire surveillance and evaluation. However, risk assessments of wildfire occurrence in high-voltage PLCs have rarely been reported thus far.

For line tripping, it means that a transmission line is tripped when it starts carrying the fault current or it gets broken. It is a protective measure which essentially isolates the faulty lines from the rest of the healthy sections. As will be discussed later in Section 2, the line tripping can be caused by wildfires because they can affect the conductor sag, as well as the conductivity between transmission lines. Among existing studies, Hu et al. [14] analyzed how line tripping is caused by a wildfire. In addition, the impact of wildfires on the line rating has been modeled by Ansari et al. [15], and their studies were focused on the development of a model for operating the power grid during the course of a progressing wildfire. However, risk assessments of line tripping, which is caused by wildfires occurring in high-voltage PLCs, have not been reported to date. It should be noted that a fire occurrence inside or near a PLC does not necessarily lead to line tripping.

In this paper, a novel model developed to assess the risk of line tripping caused by wildfire occurrence in high-voltage PLCs is presented. This model includes the wildfire risk assessment for PLCs and the estimation of the probability of line tripping when a wildfire occurs in PLCs. The former was developed based on previous works such as [10,16] but adapted to high-voltage PLC conditions. Specifically, referring to the aforementioned existing works, we first determined which kind of natural and human factors should be chosen as input variables based on their potential influences on wildfire occurrence, and how to obtain their values. Then, we considered how to use a traditional logic regression approach to obtain the risk levels of wildfire occurrence in high-voltage PLCs, and how to evaluate the performance of the developed model. The probability of line tripping caused by wildfires was also estimated with the logic regression method, but the influencing factors were selected based on the mechanism analysis of line tripping caused by wildfires. With this model, some valuable suggestions in terms of predicting wildfire occurrence in high-voltage PLCs and line tripping caused by wildfires can be provided. To the best of our knowledge, this is the first work to assess the risk of line tripping caused by wildfire occurrence in high-voltage PLCs. The outline of this paper is as follows. The configuration of the developed model is first introduced in Section 2. Next, the risk assessment of wildfire occurrence in high-voltage PLCs is described in Section 3, and the estimation of the probability of line tripping caused by wildfires is provided in Section 4. This section is followed by evaluating the developed model’s performance in Section 5, where the predictive results are compared with the actual wildfire data and line tripping events. Finally, conclusions are given in Section 6.

## 2. Description of Developed Model

**Figure 1:**
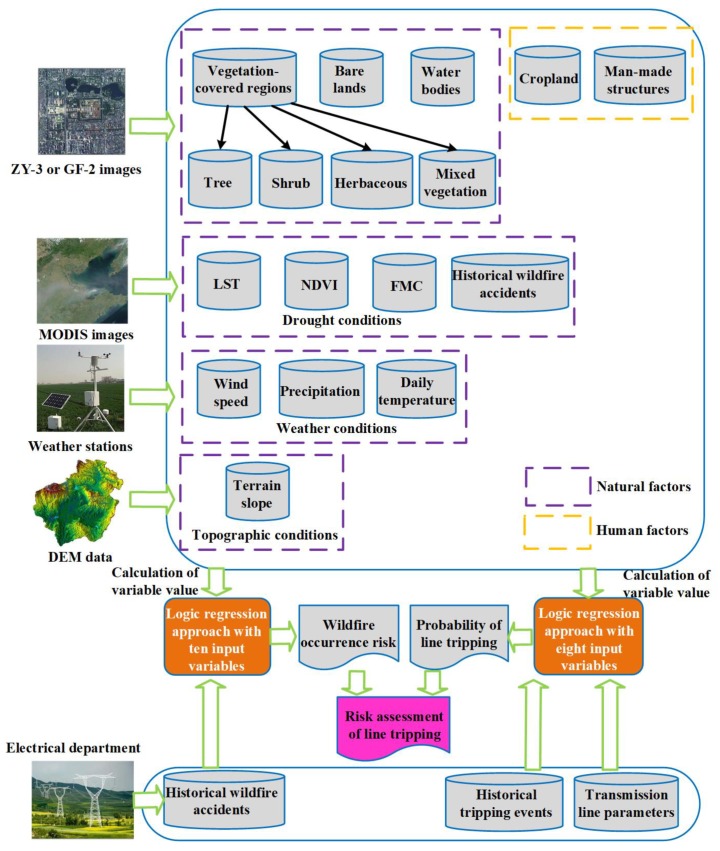
Block diagram of the model developed to assess the risk of line tripping caused by wildfire occurrence in high-voltage power line corridors. LST: land surface temperature; NDVI: normalized difference vegetation index; FMC: fuel moisture content; MODIS: Moderate Resolution Imaging Spectroradiometer; and DEM: digital elevation model.

A block diagram of the model developed to assess the risk of line tripping caused by wildfire occurrence in high-voltage PLCs is shown in Figure 1. According to the demands of the power management department, the buffer zones along the high-voltage PLCs in one Chinese province were chosen as the study area in our research, and the width of the buffer zones was set as ±1 km. Furthermore, the buffer zones along the high-voltage PLCs were evenly divided into multiple segmentations with an interval of approximately 1.5 km. In addition, the PLCs were loaded into the vector map in terms of their position vectors provided by the electrical department. It can be seen from Figure 1 that this model mainly includes the risk assessment of wildfire occurrence in high-voltage PLCs and the estimation of the probability of line tripping when a wildfire occurs inside or near a high-voltage PLC.

**Figure 2:**
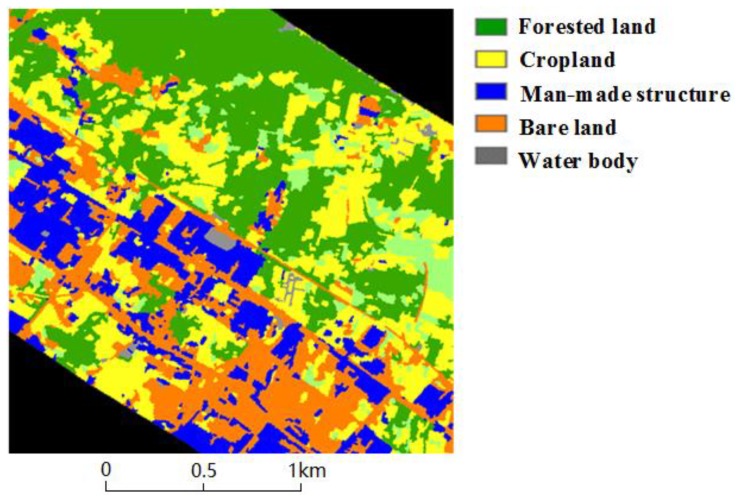
An output map of classification in one part of the high-voltage power line corridor.

Since the wildfire occurrence is related to not only natural factors but also human factors, various remotely sensed data have been employed in our research to estimate the influencing factors for the fire risk assessment. The multi-temporal data obtained from the high-resolution remote sensing satellite were adopted to classify the land-cover types in high-voltage PLCs, including the land covered by different natural vegetation, cropland, water bodies, bare lands, and man-made structures such as residential houses and graveyards. In practical operation, the high-resolution images were acquired from the ZiYuan-3 (ZY-3) satellite or the GaoFen-2 (GF-2) satellite, and a genetic algorithm similar to that described in [17] was applied to accurately classify land cover, where results with an overall accuracy of 98% can be obtained. An output map of classification in one part of the PLC is shown in Figure 2. Based on the classification results, the vegetation coverage and land cover type were chosen as two input variables for the wildfire risk assessment because fire occurrence is closely related to vegetation status at a given time [18]. Furthermore, different land cover types can also influence the wildfire occurrence significantly [19]. For example, water bodies and bare lands can decrease the probability of fire occurrence and can prevent fire spread.

In addition to high-resolution images, Moderate Resolution Imaging Spectroradiometer (MODIS) Level 1B data with a resolution of 250 m were also employed to estimate the historical ignition points and to evaluate the daily drought conditions. Here, the drought conditions include the land surface temperature (LST) that is used to describe the fuel temperature leading to fire ignition [20], the normalized difference vegetation index (NDVI) that is used to evaluate the proneness of vegetation to fire [21], and the fuel moisture content (FMC) that is one of the critical dynamic factors driving fire initiation, burning efficiency, and spread [22]. With regards to the acquisition of historical wildfire data, the MODIS data during historical observation periods were first collected, and then processed with an algorithm that uses the brightness temperatures derived from MODIS 4 and 11 µm channels. More details about this algorithm can be found in [23]. It is important to note that some historical wildfire data were acquired from the electrical department, considering that some burned areas cannot be derived from the MODIS data because of cloud cover. Before use, the MODIS data should be pre-processed, including geo-registration, de-striping, and cloud masking. As pointed out by Lozano et al. [24], since fire risk is strongly linked with fire history, the recent wildfire occurrence history should be chosen as an input variable for the wildfire risk assessment.

Fire initiation and spread are significantly influenced by weather conditions [25], therefore, the climate data collected from the weather stations in the whole province were used to provide the meteorological data such as the daily temperature, precipitation, and wind speed. The interpolation method was adopted to obtain the required meteorological data over the study area due to the sparse distribution of weather stations. In addition, the digital elevation model (DEM) data obtained from the Shuttle Radar Topography Mission (SRTM) were used to determine the topographic conditions along the high-voltage PLC because the terrain slope may affect the fire occurrence [26]. With respect to human factors, the distance between the power line and cropland, as well as the distance between the power line and man-made structures, were selected as input variables. These variables were selected because sometimes crop straws are burned after harvest [27], and many sacrificial paper and firecrackers are usually burned around man-made structures during some traditional Chinese festivals, which can lead to wildfire occurrence in high-voltage PLCs. Since the Chinese government has banned the field burning of crop straws and firecrackers in recent years, contributions of the above variables to wildfire occurrence have been reduced slightly. However, compared with natural causes, human activities still have a relatively higher contribution to wildfire accidents. When modeling fire occurrence, a logistic regression model has been regarded as a valuable tool [24,26,28,29]. Therefore, one part of the historical wildfire data was first used for training to estimate the weight of each variable, and then the logistic regression approach was exploited to perform the risk assessment of wildfire occurrence in high-voltage PLCs.

When a wildfire occurs inside or near the high-voltage PLC, it can affect the transmission line in different ways. For example, large fires can directly damage the wooden transmission towers and poles, causing the power line to completely collapse. Furthermore, the heat released by the fire can result in a rise in temperature around the conductors significantly. Such high temperature can not only lead to excessive conductor sag that decreases the safety distance of the power lines but can also result in thermal ionization to produce a large number of charged particles and can reduce the air density to lower the breakdown voltage. At the same time, the particles and ions in the smoke and soot from the fire can decrease the electric strength of the insulation strings that insulate the conductors from the tower structure, as well as that of the air which is the insulation medium between conductors or between the conductors and the ground [30]. All of these factors, in turn, could potentially lead to insulation breakdown and subsequent flashovers to give rise to the line tripping events. Therefore, when a wildfire burns inside or near the high-voltage PLC, the following various variables responsible for the occurrence of line tripping were considered in our research:

(1) Vegetation types that are not only closely related to the heat released by the fire, but are also related to the particles and ions in the smoke and soot from the fire. For example, the power line can be more easily affected by trees than shrubs when both of them are on fire. In practice, in terms of spectral signatures, the natural vegetation was further classified as tree, shrub, herbaceous, and mixed vegetation by using the deep learning method [31]. Specifically, 1 m resolution images obtained from the GF-2 satellite were employed for vegetation classification. To improve the classification accuracy, 0.5 m resolution aerial images were also adopted. In our research, we randomly chose 900 training and 300 validating samples for each class. The remaining pixels were used as a test set. During training, we used the training samples to learn weights and biases of each neuron, and used the validation samples to tune the best super-parameters such as hidden unit sizes or hidden layer numbers. The test set was used to generate final classification results. Furthermore, the accuracy of the classification results was evaluated using ground truth data, and a confusion matrix given in Table 1 was employed to show the difference between classification results and ground truth data. From Table 1, we can find that the value of overall accuracy (OA), average accuracy (AA), and Kappa coefficient was at the level of 0.9380, 0.9122, and 0.8816, respectively. Here, we only present the final results. Another paper, in which an in-depth discussion of the vegetation classification is provided, is yet to be published.
(2) Vegetation coverage was considered because the power line will be affected by wildfires over a longer period of time in the case of a high vegetation density.
(3) Fuel moisture content that influences the burning efficiency and fire spread.
(4) Weather parameters. For instance, conductor sag is influenced by the daily temperature, and fire-spread speed is influenced by the wind speed.
(5) Terrain slope that is related to the fire-spread speed.
(6) Transmission line parameters such as the distance between power lines or between the power line and the ground were considered because the breakdown voltage increases with this distance.

After variable selection, one part of the historical line-tripping data was used for training to estimate the weight of each variable, and then the logistic regression model was used to calculate the probability of line tripping when a wildfire occurs inside or near a high-voltage PLC. It is worth noting that the transmission line parameters and the historical line tripping data were provided by the electrical department. The risk assessment of line tripping in high-voltage PLCs was achieved by combining the wildfire occurrence risk with the probability of line tripping caused by wildfires, and finally, a risk map for the occurrence of line tripping caused by wildfires was plotted with ArcGIS software.

## 3. Risk Assessment of Wildfire Occurrence

In the risk assessment of wildfire occurrence, we first calculated the value of each input variable that has been listed in Section 2. Then, historical wildfire data were pre-processed and used for training to estimate the weight of each variable. Finally, a logistic regression approach was applied to estimate the probability of wildfire occurrence in high-voltage PLCs.

### 3.1. Fire-Influencing Variables

As pointed out by Zhang et al. [10], a universal criterion for selecting fire-influencing factors does not exist because of the complex relationships between fire occurrence and influencing factors. Based on the analysis of the causes of historical wildfires in high-voltage PLCs, ten fire-influencing variables (see Table 2) were selected in our research to model the wildfire occurrence in high-voltage PLCs, where these variables can be further divided into static and dynamic categories. Taking into account the data availability for dynamic variables in practice, an appropriate temporal sampling interval should be determined to achieve the required prediction accuracy. The calculation of each variable’s value is described as follows.

According to the land-cover classification results, the natural vegetation coverage in high-voltage PLCs can be calculated as follows:

(1) Vc=Va/Ta

where Va denotes the area of natural vegetation coverage, and Ta represents the total area of the study region. In addition, land cover (LC) was obtained from the fine classification of natural vegetation and was coded by numbers 1 to 7 for different land-covered regions in ArcGIS software.

Based on MODIS images, NDVI can be obtained by:

(2) $$\mathrm{NDVI}=\frac{\left(\rho_{NIR}-\rho_{R}\right)}{\left(\rho_{NIR}+\rho_{R}\right)}$$

where $\rho_{NIR}$ is the reflectivity of the near-infrared band, and $\rho_{R}$ is the reflectivity of the red band. Furthermore, LST can be retrieved from MODIS data with the method described in [32]. To reduce the number of fire-influencing variables, the dryness index TVDI was used instead, and its relationship with NDVI and LST can be written as:

(3) $$\mathrm{TVDI}=\frac{\left(\mathrm{LST}-T_{\min}\right)}{\left(T_{\max}-T_{\min}\right)}$$

where $T_{\min}=a_{1}+b_{1}(\mathrm{NDVI})$ is the minimum surface temperature and defines the wet edge, $T_{\max}=a_{2}+b_{2}(\mathrm{NDVI})$ is the maximum surface temperature and defines the dry edge, $a_{1}$ and $a_{2}$ are the intercepts for the wet and dry edges, respectively, $b_{1}$ and $b_{2}$ are the slopes for the wet and dry edges, respectively. In addition, we estimated FMC from MODIS images with the method similar to that described in [33], and derived LF using the burned areas maps that were generated with the historical wildfire data.

Climate parameter Ws was directly obtained from the meteorological data, and Percentage of precipitation anomaly (PPA) was achieved by:

(4) $$\mathrm{PPA}=\frac{\left(R_{c}-R_{h}\right)}{R_{h}}$$

where $R_{c}$ denotes the currently observed precipitation, and $R_{h}$ denotes the historic average precipitation. In addition, Ts was calculated from the DEM by using ArcGIS software, and Dc and Dh were estimated from the vector map. During the estimation of variable Dh, we only considered residential houses and graveyards based on the analysis of the causes of historical wildfires in high-voltage PLCs.

**Figure 3:**
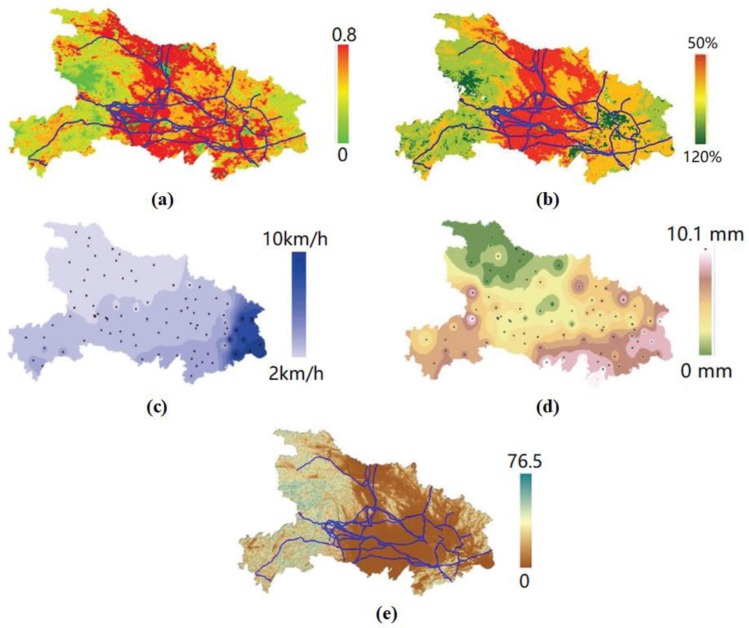
Examples of the real values of several input variables obtained in Hubei Province. (**a**) TVDI distribution in 1 October 2014; (**b**) FMC distribution in 1 October 2014; (**c**) average wind speed in 10 June 2014; (**d**) average precipitation in 10 June 2014; and (**e**) terrain slope.

Examples of the real values of several input variables that were obtained in Hubei Province are shown in Figure 3.

### 3.2. Analysis of Wildfire Occurrence Probability

The longitude, latitude, and occurrence time of historical ignition points in high-voltage PLCs were first obtained from MODIS images or gathered from the electrical department. In addition to the ignition points, the historical wildfire data also included the unburned points that were randomly selected from the same PLC at the same time. It should be noted that the number of unburned points is the same as that of historical ignition points. Then, the historical wildfire data were divided into two subsets that were used for training and validation, respectively. Here, one subset was used for model development and inner validation, and another subset was used for independent validation to test the model’s predictive capabilities under new conditions.

The logistic regression model used in the risk assessment of wildfire occurrence in high-voltage PLCs can be described as:

(5) $$\eta_{fire}=\frac{1}{\left(1+e^{-\kappa }\right)}$$

(6) $$\kappa =\kappa_{0}+\kappa_{1}x_{1}+\cdots +\kappa_{10}x_{10}$$

where $\eta_{fire}$ denotes the wildfire occurrence probability and varies from 0 to 1 in an S-shaped curve, $x_{i}(i=1,\cdots ,10)$ represent the independent fire-influencing variables, $\kappa_{0}$ is a constant, $\kappa_{i}(i=1,\cdots ,10)$ are the coefficients reflecting the contribution of the independent variable $x_{i}$ to the $\eta_{fire}$. In practical operation, $x_{1}$ denotes Vc, $x_{2}$ denotes TVDI, $x_{3}$ denotes FMC, $x_{4}$ denotes LF, $x_{5}$ denotes Ws, $x_{6}$ denotes PPA, $x_{7}$ denotes LC, $x_{8}$ denotes Ts, $x_{9}$ denotes Dc, and $x_{10}$ denotes Dh. The parameters $\kappa_{i}(i=0,1,\cdots ,10)$ were estimated by the training data, and the weight of each variable can be interpreted by the exponential function $e^{\kappa_{i}}$. In addition, the human factors considered in our research show a strong seasonality. For example, the Spring Festival occurs between January and February every year in China, the Qingming Festival occurs between March and April, and the crop straws are always burned in March, April, October, and November. Therefore, Equation (6) can be further divided as:

(7) $$\kappa_{hi}=\kappa_{0}+\kappa_{1}x_{1}+\cdots +\kappa_{10}x_{10}$$

(8) $$\kappa_{nh}=\kappa_{0}+\kappa_{1}x_{1}+\cdots +\kappa_{8}x_{8}$$

where $\kappa_{hi}$ denotes the fitting equations during the aforementioned months in which the wildfire occurrence may be caused by human activities, and $\kappa_{nh}$ denotes the fitting equations during the rest of the year. After the logistic regression analysis, according to the demands of the power management department, the wildfire occurrence risk in high-voltage PLCs was equally divided into four classes to represent the risk levels, where the fourth class represents the highest risk of fire occurrence (0.75–1.00), and the first class represents the lowest risk of fire occurrence (0–0.25).

## 4. Risk Assessment of Line Tripping

Similar to the steps in the risk assessment of wildfire occurrence, when assessing the risk of line tripping, we first calculated the value of each influencing variable that has been introduced in Section 2. Then, the historical line tripping data were pre-processed and used for training to estimate the weight of each variable. Furthermore, the logistic regression approach was employed to calculate the probability of line tripping when a wildfire occurs inside or near a high-voltage PLC. Finally, we considered the wildfire risk and the probability of line tripping caused by wildfires together to assess the risk of line tripping in high-voltage PLCs.

### 4.1. Influencing Variables of Line Tripping Caused by Wildfires

Eight influencing variables were used to model the line tripping when a wildfire occurs inside or near the high-voltage PLC. In addition to the five variables Vc, Vt, FMC, Ws, and Ts that have been analyzed in Section 2, three new variables were also employed, which include the daily temperature Td, the distance Dp between power lines, and the distance Dg between the power line and the ground. In practice, the value of Td was directly obtained from the observed meteorological data, and the values of Dp and Dg were provided by the electrical department.

### 4.2. Estimation of the Probability of Line Tripping Caused by Wildfires

Similar to the pre-processing of historical wildfire data, we first acquired the longitude, latitude, and occurrence time of historical line tripping events caused by wildfires from the electrical department, and then randomly selected the same number of normal points from the same high-voltage PLC at the same time to form the historical data of line tripping. Subsequently, the historical data of line tripping were partitioned into two subsets that were used for training and validation, respectively. Here, one subset was used for model development and inner validation, and another subset was used for independent validation to test the model’s predictive capabilities under new conditions.

The logistic regression model used in the estimation of the probability of line tripping can be described as:

(9) $$\eta_{p\_lt}=\frac{1}{\left(1+e^{-\hbar }\right)}$$

(10) $$\hbar =\hbar_{0}+\hbar_{1}y_{1}+\cdots +\hbar_{8}y_{8}$$

where the sigmoid function $\eta_{p\_lt}$ denotes the probability of line tripping when a wildfire occurs inside or near the high-voltage PLC, $y_{i}(i=1,\cdots ,8)$ represent the independent variables influencing the line tripping, $\hbar_{0}$ is the intercept of the model, and $\hbar_{i}(i=1,\cdots ,8)$ are the coefficients of input variables and can be estimated by the training data. In practical operation, $y_{1}$ denotes Vc, $y_{2}$ denotes Vt, $y_{3}$ denotes FMC, $y_{4}$ denotes Vs, $y_{5}$ denotes Td, $y_{6}$ denotes Ts, $y_{7}$ denotes Dp, and $y_{8}$ denotes Dg. Following the logistic regression analysis, to meet the demands of the power management department, the probability of line tripping was equally divided into four classes to represent the probability levels in the case of a wildfire occurrence inside or near the high-voltage PLC, where the fourth class represents the highest probability of line tripping (0.75–1.00), and the first class represents the lowest probability of line tripping (0–0.25). Furthermore, the risk assessment of line tripping $\eta_{r\_lt}$ was achieved by considering the wildfire occurrence risk and the probability of line tripping when a wildfire occurs inside or near the high-voltage PLC together, where $\eta_{r\_lt}$ is given by:

(11) $$\eta_{r\_lt}=\eta_{fire}\times \eta_{p\_lt}.$$

Finally, in terms of the demands of the power management department, the risk of line tripping caused by wildfires in high-voltage PLCs was equally divided into four classes to represent the risk levels, similar to that of wildfire occurrence described in Section 3. Moreover, the risk map of line tripping was plotted with ArcGIS software for a good visualization.

## 5. Experimental Results and Discussion

**Figure 4:**
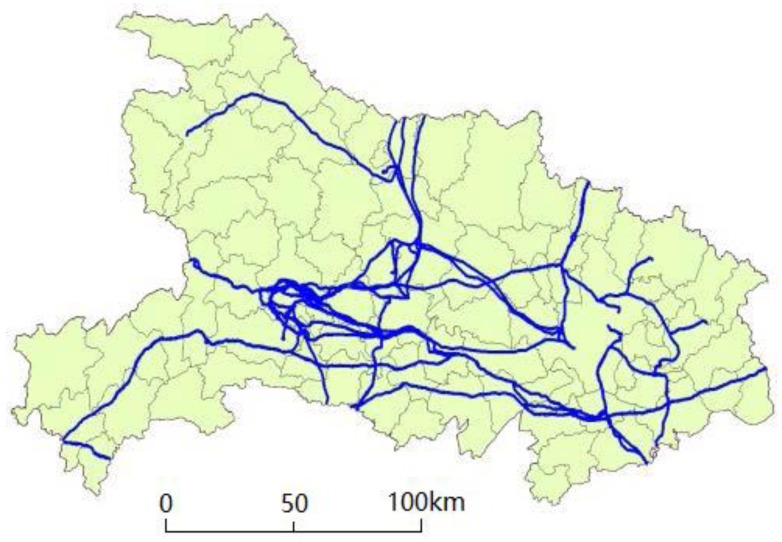
Map of Hubei Province and the distribution of ultra- and extra-high-voltage power line corridors shown in blue.

The buffer zones along the ultra- and extra-high-voltage PLCs in Hubei Province were chosen as the study area in our experiments, as shown in Figure 4. In the first experiment, a total of 350 historical ignition points recoded along the PLCs from 2008 to 2014 in the whole Hubei Province were used. Here, 200 historical ignition points were derived from the MODIS data as described in Section 2, and the rest of the historical ignition points were estimated from the data that were purchased from the State Grid Hubei Electric Power Co. Ltd. (Wuhan, China) (www.hb.sgcc.com.cn). In addition, seventy percent of the historical wildfire data were randomly selected for training, and the remaining data were used for inner validation.

Based on the above historical wildfire data, the obtained logistic regression models are shown as follows:

(12) $$\kappa_{hi}=6.21+0.43x_{1}+0.56x_{2}+0.52x_{3}+0.12x_{4}-0.05x_{5}+0.48x_{6}+0.41x_{7}-0.03x_{8}+0.87x_{9}+1.26x_{10}$$

(13) $$\kappa_{nh}=5.07+0.49x_{1}+0.82x_{2}+0.71x_{3}+0.14x_{4}-0.03x_{5}+0.66x_{6}+0.57x_{7}-0.04x_{8}.$$

As described by Zhang et al. [10], if a coefficient in the logistic regression model is positive, this means the event is more likely to occur, otherwise the odds of the event will decrease. According to Equations (12) and (13), one can find that the most significant factor was Dh during these months in which the wildfire occurrence may be caused by human activities, and the factor Dc also had a large contribution. During the rest of the year, the factor TVDI played the most important role in the wildfire occurrence. In contrast, the factors Ws and Ts had much lower contributions in our research.

**Figure 5:**
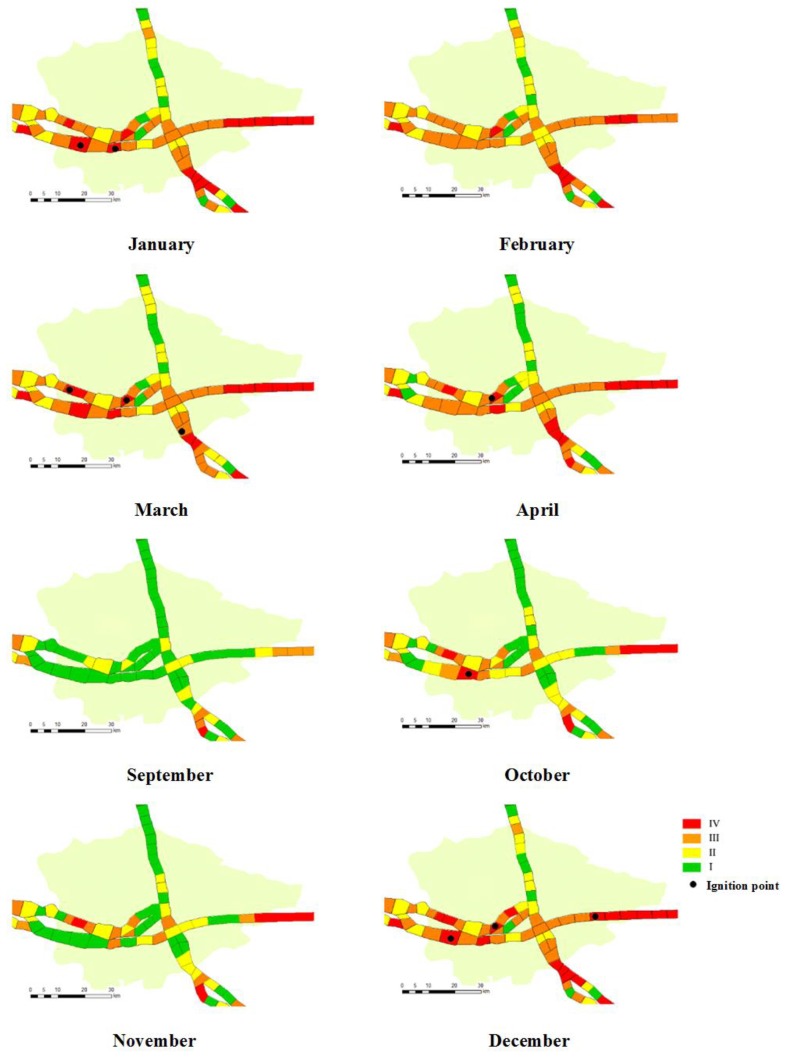
A comparison between the predictive results provided by the developed model and the actual wildfire accidents that occurred in Xianning (29.50° N, 114.19° E) in 2015.

To test the model’s predictive capabilities under new conditions, 56 ignition points recorded from 2015 to 2016 were used for independent validation. Figure 5 shows a comparison between the predictive results provided by the developed model and the actual wildfire accidents that occurred in Xianning (29.50° N, 114.19° E) in 2015, where the buffer zones along the high-voltage PLCs were divided into a total of 67 segmentations. Since the historical wildfire accidents recorded from 2008 to 2014 mostly occurred between January and April and between October and December, we provide the comparison results in these months.

As shown in Figure 5, a higher risk of wildfire occurrence can be found between January and April and between October and December because of the drought conditions and human activities. This result is consistent with the statistical characteristics of historical wildfire occurrence. Furthermore, most of the actual ignition points are located at the segmentations having the highest risk of wildfire occurrence, except for one ignition point that is located at the segment having the risk level of 0.5–0.75. This result is because the probability of wildfire occurrence was predicted for each month rather than each day. Hence, the vegetation characteristics and the weather conditions may vary daily. In addition, we can see from Figure 5 that the line segment located at the right-hand side shows a very high risk throughout the observed period due to the high vegetation density. In contrast, the line segment located at the upper left side shows a low risk during the observed period because this region mainly consists of water bodies. Therefore, it can be found that the predictive results of wildfire occurrence in high-voltage PLCs provided by the developed model are in good agreement with the actual wildfire accidents.

To further evaluate the prediction accuracy of the proposed model when it was applied to the risk assessment of wildfire occurrence, the cumulative frequency was adopted and calculated by counting the number of real ignition points that fall within each risk level generated by the proposed model. Table 3 shows the final results. One can see that 92.86% (i.e., 52/56) of actual ignition points fall within the highest risk of fire occurrence (0.75–1.00). Moreover, a false alarm rate of 13.33% (i.e., 8/(52 + 8)) were obtained. It should be noted that a wildfire will not occur in the case of no ignition source, although the fire risk is very high. In addition, one can find that the false alarm given in Table 3 is lower than that shown in Figure 5, because the probability of wildfire occurrence given in Table 3 was predicted for each day rather than each month.

In the second experiment, a total of 30 historical line tripping events, which were caused by wildfires in high-voltage PLCs and recoded from 2008 to 2014 in the whole Hubei Province, were used. Here, all historical line tripping data were also purchased from the State Grid Hubei Electric Power Co. Ltd. (www.hb.sgcc.com.cn). Furthermore, eighty percent of the historical tripping data were randomly selected for training, and the remaining data were used for inner validation. According to the above historical wildfire data, the obtained logistic regression model is shown as follows:

(14) $$\hbar =5.42+0.47y_{1}+0.49y_{2}+0.45y_{3}-0.02y_{4}+0.55y_{5}-0.05y_{6}+1.33y_{7}+1.17y_{8}.$$

From Equation (14), one can see that the most significant factor was Dp, and the factor Dg also had a large contribution. In addition, factors Vc, Vt, FMC, and Td had similar contributions. In contrast, Ws and Ts had much lower contributions.

**Figure 6:**
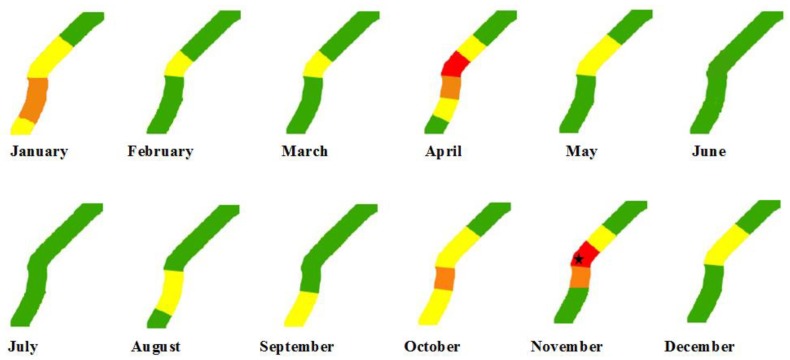
A comparison between the predictive results provided by the developed model and the actual line tripping events that occurred in Huanggang (30.27° N, 114.52° E) on the first day of each month in 2015, where the black star denotes the location of an actual line tripping event.

In addition, 16 actual line tripping events, which were caused by wildfires and recorded from 2015 to 2016, were used for independent validation. It is worth noting that since there was no historical tripping event corresponding to these experimental datasets shown in Figure 5, a different place was chosen for following analysis. Figure 6 shows a comparison between the predictive results provided by the developed model and actual line tripping events that occurred in Huanggang (30.27° N, 114.52° E) on the first day of each month in 2015, where the buffer zones along the high-voltage PLCs were divided into a total of seven segmentations.

As shown in Figure 6, a higher risk of line tripping can be found in January, April, October, and November. This result is almost consistent with the statistical characteristics of historical line tripping events, except for February, March, and December because strong precipitation during the above three months decreased the risk of wildfire occurrence and, hence, the risk of line tripping caused by wildfires. Furthermore, the location of an actual line tripping event was located in the segment having the highest risk of line tripping. However, we should note that the line tripping did not occur at the segment having the highest risk of line tripping in April, although a wildfire occurrence was found. In addition, it can be seen from Figure 6 that the line segment located in the middle position always shows a very high risk throughout the observed period due to the high tree cover. In contrast, the line segment located at the upper right side shows a low risk during the observed period, because this region mainly consists of bare lands. Therefore, it can be found that the predictive results of line tripping caused by wildfires in high-voltage PLCs are consistent with the actual line tripping events.

To further evaluate the prediction accuracy of the proposed model when it was applied in the risk assessment of line tripping, the cumulative frequency was adopted and calculated by counting the number of real tripping events that fall within each risk level generated by the proposed model. Table 4 shows the final results. One can see that 93.75% (i.e., 15/16) of actual tripping events fall within the highest risk of line tripping (0.75–1.00). Moreover, a false alarm rate of 16.67% (i.e., 3/(15 + 3)) can be obtained. It should be noted that a line tripping caused by wildfires may not occur if there is no ignition source or some actions described in [15,30] are taken by the power system operator, although the risk level of line tripping is very high.

## 6. Conclusions

This paper presented a novel model developed to assess the risk of line tripping caused by wildfire occurrence in high-voltage PLCs. The experimental results have preliminarily demonstrated the reliability of this model when it is applied to assess the risk of wildfire occurrence and the resulting line tripping in high-voltage PLCs. Therefore, it can be used for providing some useful suggestions to the electrical department, such as removing vegetation from the regions with a high risk. In future work, due to the high dependence of wildfire occurrence and line tripping on the type and height of vegetation, we will use multi-temporal and multi-source remote-sensing data to better classify the vegetation in high-voltage PLCs, such as light detection and ranging (LIDAR) data [34,35]. At the same time, the vegetation biomass will be included in the risk assessment of wildfire occurrence, as recommended by Sannier et al. [36]. Furthermore, more historical line tripping events caused by wildfires will be gathered for training and validation, and other methods such as the artificial neural network will be used to obtain the risk levels. In addition, the probability of wildfire occurrence and the resulting line tripping will be estimated for a time interval shorter than a day to improve the predictive accuracy of the risk assessment. Also, we will adjust the parameters of the developed model, and apply it to perform the risk assessment of wildfire occurrence and line tripping for high-voltage PLCs in other Chinese provinces.

## Figures and Tables

**Table 1:** Confusion matrix of vegetation classification.

Vegetation Types	Tree	Shrub	Herbaceous	Mixed Vegetation	
Tree	2612	83	6	101	2802
Shrub	68	3236	41	132	3477
Herbaceous	8	59	2766	98	2931
Mixed vegetation	172	202	157	2989	3520
Total number of test set	2860	3580	2970	3320	12,730

**Table 2:** Variables used to model wildfire occurrence in high-voltage power line corridors.

Variable	Variable Description (Unit)
Dynamic category:	
Vc	Natural vegetation coverage (percent)
TVDI	Temperature vegetation dryness index
FMC	Fuel moisture content (percent)
LF	Number of years since the last fire event
Ws	Wind speed (km/hour)
PPA	Percentage of precipitation anomaly (percent)
Static category:	
LC	Land cover types:
	Code 1—Tree
	Code 2—Shrub
	Code 3—Herbaceous
	Code 4—Mixed vegetation: tree and shrub
	Code 5—Mixed vegetation: shrub and herbaceous
	Code 6—Water body
	Code 7—Bare land
Ts	Terrain slope (percent)
Dc	Distance to the nearest cropland (km)
Dh	Distance to the nearest man-made structures (km)

**Table 3:** The cumulative frequency used to account for the number of real ignition points that fall within each risk level, and the false alarm rate used to account for the case where a higher risk of fire occurrence was predicted but no actual fire occurred.

Risk Level	Cumulative Frequency	False Alarm Rate
0.75–1.00	52	13.33%
0.50–0.75	4	/
0.25–0.50	0	/
0–0.25	0	/

**Table 4:** The cumulative frequency used to account for the number of real tripping events that fall within each risk level, and the false alarm rate used to account for the case where a higher risk of line tripping was predicted but no actual line tripping occurred.

Risk Level	Cumulative Frequency	False Alarm Rate
0.75–1.00	15	16.67%
0.50–0.75	1	/
0.25–0.50	0	/
0–0.25	0	/

## References

[1] Huang D., Shu Y., Ruan J., Hu Y. (2009). Ultra high voltage transmission in China: Developments, current status and future prospects. Proc. IEEE.

[2] Cao S., Chen L., Yu X. (2009). Impact of China’s grain for green project on the landscape of vulnerable arid and semi-arid agricultural regions: A case study in northern Shaanxi Province. J. Appl. Ecol..

[3] Zhang Y., You F., Chen H., Zhang L., Zhu J., Zhou J. (2011). Statistical analysis of wildfire accidents inducing flashover of transmission lines. Fire Sci. Technol..

[4] Hu Y., Liu K., Wu T., Liu Y., Su Z. (2014). Analysis of influential factors on operation safety of transmission line and countermeasures. High Volt. Eng..

[5] Chuvieco E., Aguado I., Yebra M., Nieto H., Salas J., Martin M.P., Vilar L., Martinez J., Martin S., Ibarra P. (2010). Development of a framework for fire risk assessment using remote sensing and geographic information system technologies. Ecol. Modell..

[6] Oliveira S., Oehler F., San-Miguel-Ayanz J., Camia A., Pereira J.M.C. (2012). Modeling spatial patterns of fire occurrence in Mediterranean Europe using Multiple Regression and Random Forest. For. Ecol. Manag..

[7] Vadrevu K.P., Eaturu A., Badarinath K.V.S. (2010). Fire risk evaluation using multicriteria analysis—A case study. Environ. Monit. Assess..

[8] Yu B., Chen F., Li B., Wang L., Wu M. (2017). Fire risk prediction using remote sensed products: A case of Cambodia. Photogramm. Eng. Remote Sens..

[9] Satir O., Berberoglu S., Donmez C. (2016). Mapping regional forest fire probability using artificial neural network model in a Mediterranean forest ecosystem. Geomat. Nat. Hazards Risk.

[10] Zhang H., Han X., Dai S. (2013). Fire occurrence probability mapping of northeast China with binary logistic regression model. IEEE J. Sel. Top. Appl. Earth Obs. Remote Sens..

[11] Chowdhury E.H., Hassan Q.K. (2015). Operational perspective of remote sensing-based forest fire danger forecasting systems. ISPRS J. Photogramm. Remote Sens..

[12] Lu J., Yang L., Zhang H.X., Li B., Lin H. Research on large-area fire monitoring and warning system for power grid. Proceedings of the 2012 International Conference on Systems and Informatics.

[13] Chen X., Li T., Ruan L., Xu K., Huang J., Xiong Y. Research and application of fire risk assessment based on satellite remote sensing for transmission line. Proceedings of the World Congress on Engineering and Computer Science.

[14] Hu X., Lu J., Zeng X., Zhang H. (2010). Analysis on transmission line trip caused by mountain fire and discussion on tripping preventing measures. J. Electr. Power Sci. Technol..

[15] Ansari B., Mohagheghi S. (2015). Optimal energy dispatch of the power distribution network during the course of a progressing wildfire. Int. Trans. Electr. Energy Syst..

[16] Eskandari S., Chuvieco E. (2015). Fire danger assessment in Iran based on geospatial information. Int. J. Appl. Earth Obs. Geoinform..

[17] Tseng M.H., Chen S.J., Hwang G.H., Shen M.Y. (2008). A genetic algorithm rule-based approach for land-cover classification. ISPRS J. Photogramm. Remote Sens..

[18] Maselli F., Rodolfi A., Bottai L., Romanelli S., Conese C. (2000). Classification of Mediterranean vegetation by TM and ancillary data for the evaluation of fire risk. Int. J. Remote Sens..

[19] Ardakani A.S., Zoej M.J.V., Mohammadzadeh A., Mansourian A. (2011). Spatial and temporal analysis of fires detected by MODIS data in northern Iran from 2001 to 2008. IEEE J. Sel. Top. Appl. Earth Obs. Remote Sens..

[20] Guo G., Zhou M. (2004). Using MODIS land surface temperature to evaluate forest fire risk of northeast China. IEEE Geosci. Remote Sens. Lett..

[21] Maselli F., Romanelli S., Bottai L., Zipoli G. (2003). Use of NOAA-AVHRR NDVI images for the estimation of dynamic fire risk in Mediterranean areas. Remote Sens. Environ..

[22] Chuvieco E., Gonzalez I., Verdu F., Aguado I., Yebra M. (2009). Prediction of fire occurrence from live fuel moisture content measurements in a Mediterranean ecosystem. Int. J. Wildland Fire.

[23] Justice C.O., Giglio L., Korontzi S., Owens J., Morisette J.T., Roy D., Descloitres J., Alleaume S., Petitcolin F., Kaufman Y. (2002). The MODIS fire products. Remote Sens. Environ..

[24] Lozano F.J., Suarez-Seoane S., de Luis E. (2007). Assessment of several spectral indices derived from multi-temporal Landsat data for fire occurrence probability modelling. Remote Sens. Environ..

[25] Zhang Z.X., Zhang H.Y., Zhou D.W. (2010). Using GIS spatial analysis and logistic regression to predict the probabilities of human-caused grassland fires. J. Arid Environ..

[26] Preisler H.K., Brilinger D.R., Burgan R.E., Benoit J.W. (2004). Probability based models for estimation of wildfire risk. Int. J. Wildland Fire.

[27] Zhang Z., Yu H., Hu Z., Zhou B., Han C. Agricultural burning detection using remote sensing: A case study in Zhejiang Province, China. Proceedings of the 4th International Conference on Bioinformatics and Biomedical Engineering.

[28] Verbesselt J., Somers B., van Aardt J., Jonckheere I., Coppin P. (2006). Monitoring herbaceous biomass and water content with SPOT VEGETATION time-series to improve fire risk assessment in savanna ecosystems. Remote Sens. Environ..

[29] Chang Y., Zhu Z., Bu R., Chen H., Feng Y., Li Y., Hu Y., Wang Z. (2013). Predicting fire occurrence patterns with logistic regression in Heilongjiang Province, China. Landsc. Ecol..

[30] Choobineh M., Ansari B., Mohagheghi S. (2015). Vulnerability assessment of the power grid against progressing wildfires. Fire Saf. J..

[31] Chen Y., Lin Z., Zhao X., Wang G., Gu Y. (2014). Deep learning-based classification of hyperspectral data. IEEE J. Sel. Top. Appl. Earth Obs. Remote Sens..

[32] Wan Z., Zhang Y., Zhang Q., Li Z.L. (2004). Quality assessment and validation of the MODIS global land surface temperature. Int. J. Remote Sens..

[33] Yebra M., Chuvieco E., Riario D. (2008). Estimation of live fuel moisture content from MODIS images for fire risk assessment. Agric. For. Meteorol..

[34] Mills S.J., Castro M.P.G., Li Z.R., Cai J.H., Hayward R., Mejias L., Walker R.A. (2010). Evaluation of aerial remote sensing techniques for vegetation management in power-line corridors. IEEE Trans. Geosci. Remote Sens..

[35] Matikainen L., Lehtomaki M., Ahokas E., Hyyppa J., Karjalainen M., Jaakkola A., Kukko A., Heinonen T. (2016). Remote sensing methods for power line corridor surveys. ISPRS J. Photogramm. Remote Sens..

[36] Sannier C.A.D., Taylor J.C., Du Plessis W. (2002). Real-time monitoring of vegetation biomass with NOAA-AVHRR in Etosha National Park, Namibia, for fire risk assessment. Int. J. Remote Sens..

